# Ophthalmic Therapeutics

**Published:** 1882-02

**Authors:** 


### BOOK NOTICES, REVIEWS, ETC.

Ophthalmic Therapeutics. By *Geo. S. Norton, M. D.*, Professor of Ophthalmology in the College of the N. Y. Ophthalmic Hospital, etc., etc. With an Introduction by Prof. T. F. Allen, M. D. 2nd edition, re-written and revised, with copious additions, 342 pp., 8vo. Price $2.50. Boericke & Tafel. 1882.

Another edition of this well-known work being demanded, is sufficient evidence that it is appreciated by the profession, and it may be accepted as being well worthy of appreciation from the signs of painstaking labor, particularly evident throughout part first. The author states in the preface that part first has been wholly re-written and several new remedies added, and that part second has been thoroughly revised and copious additions made. Part first, as in the first edition, contains the symptomatology of the various remedies from *Acetic acid* to *Zincum*, and with but few exceptions should command hearty praise. In viewing part second we find several very un-homœopathic prescriptions advised, and instance this, for the treatment of blepharitis ciliaris, at page 207: R. *Hydrarg. Oxyg. flav.*, gr. ij., *Vaseline*, ℥ ij.; and on the same page: R. *Liq. Hydrarg. Nit.*, gtt., iij., *Ol. Morrhuæ*, ℥ ij. On turning to Prof. Allen's introduction, we read: "While there is no doubt that the conditions of the eye, in diseases of that organ, is a most important factor in the selection of the remedy, still we must not forget that eye diseases are often, perhaps generally, the expression of a general cachexia, the remedy for which can only be found by a close examination of the whole individual." Now, we desire to ask: Who, that has had any experience in practising genuine homœopathy, will truthfully admit that idiopathic eye affections are not always "the expression of a general cachexia?" (Even the regulars acknowledge this.) And being such, how unnecessary to resort to local treatment, which may do harm!! While a student at college we frequently witnessed the result of such treatment at the hands of allopaths; the danger and uselessness of such methods, so far as permanent cures were concerned, were indelibly impressed upon us: and now to find such recommendations, especially as they follow such excellent and copious symptomatology as is given in part first of this work, fills us with surprise. At p. 224 we find advised for catarrhal conjunctivitis, because "in conjunctivitis after the acute symptoms have subsided, we sometimes find the inflammation will come to a stand-still, notwithstanding our most careful selection of remedies": R. *Zinci Sulph.*, gr. ij., *Sodium Chlorid.* gr. iv., *Aqua dist.* ℥ iv. Such treatment is likely to start it going again! We are even more surprised as we turn to this, for the best old-school authorities would not resort to such a lawless method. We know that comparisons are said to be "odious," but we cannot refrain from comparing this with one of the best authors of the old-school.

On turning to Dr. Sœlberg Wells' "Treatise on Diseases of the Eye," we find: "The prognosis of catarrhal ophthalmia is favorable, for the affection is very amenable to treatment. The milder forms generally run their course in a few days, the more severe in two or three weeks. * * * The treatment must vary according to the stage and the severity of the disease. If the eye is very irritable and there is much photophobia, lachrymation and ciliary neuralgia, accompanied by conjunctival and marked sub-conjunctival injection, astringent lotions should be carefully avoided, as they would increase the irritability, or might even set up inflammation of the cornea or iris." Notwithstanding in a later stage of the disease the old-school author advises the use of mild astringents, one can readily see that for a professed homœopath to make such recommendations, for any stage of a malady that is so "amenable to treatment," by a poorly armed allopath, is a manifestation of lack of adherence to principle, and those who have had but little experience in treating affections of the eyes should be warned of the danger that is possible in resorting to such treatment. In justice, we should find quotation marks in many passages of this part of the work, but they are missing. If any one wishes to know more and better treatment of the kind, we advise him to turn at once to Dr. Wells' work. With equal force the same remarks apply to the un-homœopathic treatment of purulent ophthalmia. On turning to *Arg. nit.* in part first, the author says: "I do believe there is no need of cauterization with it, *except in the gonorrhœal form of purulent conjunctivitis*" (the italics are the author's). Now we do know from experience that even in the worst forms of gonorrhœal ophthalmia it is superfluous to use *Arg. nit.*, or any other substance for cauterizing purposes. With perfect cleanliness, best obtained by using hot water and the indicated homœopathic remedy, one need have no fear of the result; and this we venture after treating several cases with success, even in our early days of practice. Here again, it may readily be seen that recourse to un-homœopathic treatment is needless. Are cold applications, as the author advises, to inflamed parts, homœopathic and best? We answer, decidedly, no. Did we not know that this comes from one who is a professor in the N. Y. Homœopathic Oph. Hosp., we could readily believe, when we see him advising cold compresses to the inflamed eye, that he belonged to the "regulars," except that the "regulars" *now* use warm, not cold, applications. We can here again speak from experience, and say that heat is always the best to apply to any acute inflammation—sometimes moist, at others, dry. The later works of the allopaths acknowledge also this. What we have said of the treatment of these affections is applicable to all other diseases of the eye: in no case is it necessary to step aside from a guide, our law, and flounder in darkness by resorting to expedients. Let us adhere to "that which makes law necessary." We regret that part first, so admirable, with but few exceptions, should be so marred by being tacked to part second, so anything but admirable in the parts in which superannuated treatment is advised.

